# A Novel HPLC-Assisted Method for Investigation of the Fe^2+^-Chelating Activity of Flavonoids and Plant Extracts

**DOI:** 10.3390/molecules191118296

**Published:** 2014-11-10

**Authors:** Daniil N. Olennikov, Nina I. Kashchenko, Nadezhda K. Chirikova

**Affiliations:** 1 Institute of General and Experimental Biology, Siberian Division, Russian Academy of Science, Sakh’yanovoy Str., 6, Ulan-Ude 670047, Russia; E-Mail: ninkk@mail.ru; 2 Department of Biochemistry and Biotechnology, North-Eastern Federal University, 58 Belinsky Str., Yakutsk 677-027, Russia; E-Mail: hofnung@mail.ru

**Keywords:** Fe^2+^-chelating activity, HPLC, flavonoids, antioxidant activity, *Scutellaria baicalensis*

## Abstract

Flavonoids are a class of natural phenolic compounds that show antioxidant properties. Besides the known mechanisms of action of flavonoids (binding/inactivation of free radicals and other reactive oxygen species) that determine this effect, an important factor is their ability to bind transition metal ions. In this paper, we used a HPLC method with a prechromatographic reaction of a sample with Fe^2+^ ions (FeCA-HPLC) to characterize the Fe^2+^-chelating properties of individual compounds, their mixtures, and plant extracts. Using two classes of flavonoids (flavones, flavonols) the ability of compounds to bind Fe^2+^ ions due to a number of structural features of the compounds was shown. If the compounds possessed Fe^2+^-chelating properties, the decrease in the area of the chromatographic peaks on the chromatogram was marked. By comparing the resulting chromatogram with that of the untreated sample, it was possible to estimate the value of the effect. Application of this method for the analysis of plant extracts representing a mixture of substances allows determination of the compounds that have the greatest influence on the Fe^2+^-chelating activity.

## 1. Introduction

Transition metals play a major role in the generation of reactive oxygen species (ROS) in organisms. The most common ROS include the superoxide anion (O_2_^•−^), the hydroxyl radical (OH^•^), singlet oxygen (O_2_), and hydrogen peroxide (H_2_O_2_). Superoxide anion is readily produced through the one-electron reduction of oxygen by the Fe^2+^ ion, and is largely dismuted into hydrogen peroxide by enzymatic and nonenzymatic mechanisms [1]. Hydrogen peroxide is further converted to a hydroxyl radical by the Fenton reaction, which requires the ions of Cu^2+^ or Fe^2+^ [2]. The perferryl ion (Fe^2+^-O_2_) forms after binding Fe^2+^, which is reduced with molecular oxygen [3,4]. ROS are potent oxidizing and reducing agents that directly damage cellular membranes by lipid peroxidation [5]. 

The biomedical literature is full of claims that free radicals and other reactive species are involved in human diseases. They have been implicated in over 150 disorders, including rheumatoid arthritis and haemorrhagic shock [6,7], cardiomyopathy and intestinal ischaemia [8], AIDS [9] and even male-pattern baldness [10]. 

Antioxidants are compounds that protect cells against the damaging effects of ROS, including flavonoids, which are natural compounds with antioxidant properties. Flavonoids are secondary metabolites in all higher plant genera. They have been shown in recent years to be of vital significance to mankind due to their various pharmacological properties [11,12,13]. A great deal of antioxidant investigations have verified the ability of flavonoids to scavenge free radicals [14,15,16]. Flavonoids act as natural antioxidants in the human body through various mechanisms, with an emphasis on suppressing ROS formation by inhibiting enzymes, the direct scavenge of ROS, and the regulation or protection of antioxidant defences. Besides the known mechanisms of flavonoids (binding/inactivation of free radicals and other ROS) that determine this effect, an important factor is their ability to bind transition metal ions. Flavonoids can coordinate with transition metals to catalyse electron transport and promote free radical scavenge [17]. The mechanism of metal ion chelation by polyphenols arouses significant interest today. Different classes of phenolic compounds have been investigated for their ability to chelate metal ions, including phenolic acids [18,19,20], flavonoids [21,22,23] and chalcones [22].

Recently, particular attention has been devoted to the creation of convenient and reliable methods for studying the antioxidant activity of plant extracts. A relatively new development in this field is the HPLC-assisted techniques, combining chromatographic analysis and pre/post-column derivatization of the sample with the use of various agents, such as DPPH-HPLC or ABTS-HPLC. Given the special interest in investigation of metal-chelating activity, a promising task is the creation of correct, rapid and inexpensive methods that integrate pre-column derivatization and advantages of HPLC for the determination of individual compounds in *on-line* condition. 

This work aimed to develop a new and convenient HPLC-assisted method that uses a prechromatographic reaction of a sample with Fe^2+^ ions as a tool for investigation of Fe-chelating activity of individual compounds and plant extracts.

## 2. Results and Discussion

### 2.1. Preliminary Clarification of Experimental Design 

In order to reveal the effectiveness of the proposed technique investigating Fe^2+^-chelating activity (Fe-CA), the experimental part of the work was carried out on samples of standard compounds during the first stage of the study. Given the suggestion by various researchers of the leading role of phenolic compounds in the manifestation of Fe-CA, representatives of flavonoids (we used commercially available samples) were selected. The general scheme of analysis consists of preparing solutions of the compounds at a certain concentration, processing the sample with Fe^2+^ salt, incubation, filtration and then the HPLC study. A solution of a sample treated in a similar manner except that the solution of Fe^2+^ salt was substituted for distilled water was used as a reference.

Based on the data regarding the different abilities of the compounds to be solubilised in solvents that are used for working with phenolic compounds, we proposed DMSO as a universal solvent. In preliminary investigations, the presence of this solvent in the incubation medium did not affect the results of the analysis; moreover, the presence of DMSO allows analysis by HPLC.

FeSO_4_∙7H_2_O was used as the Fe^2+^ salt, as it is soluble in water and does not precipitate when mixed with DMSO. The final concentration of the Fe^2+^ ions in the 2% incubation medium creates an excess of ions, which in turn allows a more complete chelation. Because the optimal pH for complex formation is around 6.0, this level was used for process solutions [21]. 

The incubation time selected was 30 min. During this time, changes in the residual concentration of the test compounds did not occur. The temperature of the incubation medium was 37 °C as being the most close to physiological body temperature. 

It should be noted that excess FeSO_4_ in the sample does not affect the quality of chromatographic analysis by HPLC. Given the high hydrophilicity of this salt on reversed-phase sorbents, which are traditionally used for the analysis of phenols, residual amounts eluted with the dead volume of the column.

Efficacy of chelation was assessed by the value of Δ*S* (percentage), which is the reduction of the peak area of the compound after the reaction of Fe^2+^ ions with respect to the sample without the introduction of metal ions. The results are presented in the form of two superposed chromatograms, including chromatogram before treatment and inverted chromatogram after treatment to improve the visual perception of chelating effectiveness.

### 2.2. Characterization of Fe-CA of Flavonoid Standards by HPLC Using a Prechromatographic Reaction with Fe^2+^ Ions

Our study of Fe-CA of flavonoids was conducted on representatives of two structural types—flavones and flavonols (flavon-3-ols) whose derivatives are the most frequently identified in the plant world [24]. During the first stage, we studied the possibility of using the previously described hypothesis on a sample of the compound having notoriously high chelating activity. Among the flavonoids, flavone baicalein is one of the well-known chelators with high binding capacity of Fe^2+^ ions, and has been the subject of numerous studies.

**Figure 1:**
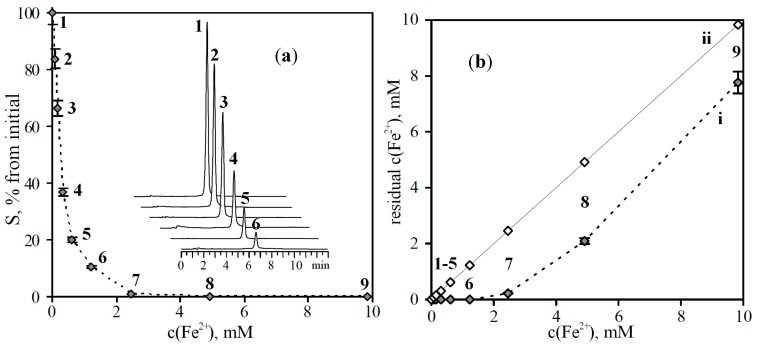
(**a**) Reduction of peak areas (S) of baicalein after prechromatographic reaction with different concentration of Fe^2+^ ions. On cut—chromatograms of baicalein samples before (**1**) and after prechromatographic reaction with Fe^2+^ ions (**2**–**6**). (**b**) Residual concentration of Fe^2+^ ions after reaction of baicalein with Fe^2+^ ions *versus* initial concentration of Fe^2+^ ions (**i**). On **ii**—hypothetical curve in case of absence of chelation processes.

For the implementation of the task, a solution of baicalein with a concentration of 1.48 mM (*ca*. 400 μg/mL) was prepared, and solutions of FeSO_4_ at different concentrations (0.154–19.656 mM) were introduced into it in ratio 1:1. In the presence of a chelating effect in the test samples of baicalein, a decrease of the peak area of the compound on the chromatograms should be observed in the course of the rise in the concentration of Fe^2+^ ions. This relationship has been confirmed experimentally, namely, the peak area of baicalein in the sample without Fe^2+^ ions was approximately 30,000 AU, and this value gradually decreased to a value that was less than the limit of detection (the compound was not detected at a concentration of Fe^2+^ more than 2.5 mM) (Figure 1a). 

c(Fe^2+^), mM: 1. 0; 2. 0.077; 3. 0.154; 4. 0.307; 5. 0.614; 6. 1.228; 7. 2.457; 8. 4.914; 9. 9.828.

It is known that flavonoids in presence of metal ions undergo an autoxidation process with formation of *p*-quinonemethide intermediates in neutral solution with production of hydrogen peroxide [25]. The influence of Fe^2+^ ions on this process is less extensive *versus* Cu^+^ or Cu^2+^ ions that resulted to low rate of intermediates formation.

To prove the existence of the chelation process in the reaction mixture, we determined the residual concentration of Fe^2+^ ions after reaction of baicalein solutions with Fe^2+^ ions. The results have shown that working solutions with a concentration of Fe^2+^ ions up to 1.23 mM are characterized by undetectable levels of residual Fe^2+^ ions (Figure 1b, curve i). The sharp increase of the residual concentration of Fe^2+^ ions is observed after application of the solutions with a concentration more 1.23 mM. With the absence of chelation process the curve would have been a line (Figure 1b, curve ii).

Thus, by the example of a compound having chelating ability, the efficacy of the hypotheses of using HPLC as a tool for the detection of compounds that have the ability to bind metal ions was demonstrated.

**Figure 2:**
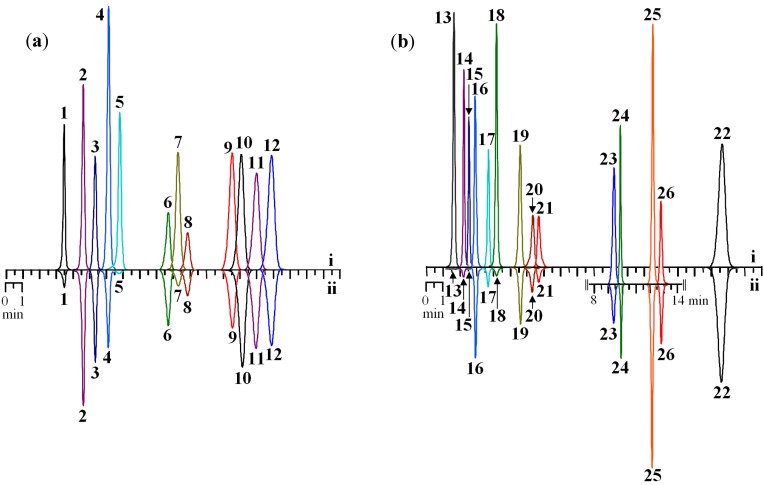
HPLC chromatograms of selected flavonoids standards (125 μg/mL) before (**i**) and after prechromatographic reaction with Fe^2+^ ions (**ii**). Samples: (**a**) flavones; (**b**) flavonols (on cut—chromatogram of flavonol glycosides). Detector λ—270 nm.

Given the huge variety of the structural types of flavonoids, we selected a few “basic” compounds with different types of replacement in the main flavonoid framework. The representatives of the studied groups of flavonoids showed different efficacy of chelation of Fe^2+^ ions (Figure 2).

Baicalein (97.35%), scutellarein (100%) and 6-hydroxyluteolin (100%) were the most effective chelators of Fe^2+^ ions from the flavone series (Table 1). Unsubstituted flavone lacks the ability to bind metal ions. The insertion of two hydroxyl groups at positions C-5 and C-7 of the A-ring (chrysin) increases activity by 11.87%. Hydroxyl substitution at position C-7 of the methoxyl group (tectochrysin) slightly reduces the efficiency index, while the substitution of both groups (dimethoxychrysin) leads to a completely inactive compound. 

1. luteolin; 2. apigenin; 3. chrysoeriol; 4. norwogonin; 5. baicalein; 6. sinensetin; 7. oroxylin A; 8. eupatorin; 9. wogonin; 10. chrysin; 11. acacetin; 12. genkwanin; 13. quercetagetin; 14. patuletin; 15. myricetin; 16. morin; 17. fisetin; 18. quercetin; 19. kaempferol; 20. isorhamnetin; 21. rhamnetin; 22. galangin; 23. isomyricitrin; 24. isoquercitrin; 25. spiraeoside; 26. astragalin.

The presence in the A-ring of flavone of three ordinary (C-5, C-6, C-7) hydroxyl groups (baicalein) sharply increases the activity up to 97.35%, the substitution one of them at C-6 (oroxylin A) leads to a reduction in activity (82.86%). The presence of 5,7,8-trihydroxy-type substitution (norwogonin) also increases the activity of the compound to 68.25% compared with the 5,7-dihydroxy-analogue (chrysin); the insertion of methoxyl at C-8 (wogonin) affects the reduction of activity (47.46%).

**Table 1:** Reduction of peak areas (Δ*S*) of flavones and flavonols after prechromatographic reaction with Fe^2+^ ions.

Compound	Substituents Positions			Δ*S*, %
	OH	MeO	Glucosyl	
*Flavones*				
Flavone	-	-	-	0
Dimethoxychrysin	-	5,7	-	0
Sinensetin	-	5,6,7,3',4'	-	0
Tectochrysin	5	7	-	9.30 ± 0.21 ^a^
Acacetin	5,7	4'	-	10.23 ± 0.23 ^b^
Chrysin	5,7	-	-	11.87 ± 0.25 ^c^
Chrysoeriol	5,7,4'	3'	-	17.83 ± 0.37 ^d^
Apigenin	5,7,4'	-	-	18.01 ± 0.39 ^ce^
Genkwanin	5,4'	7	-	19.02 ± 0.41 ^de^
Eupatorin	5,3'	6,7,4'	-	24.75 ± 0.47 ^f^
Wogonin	5,7	8	-	47.46 ± 0.85 ^i^
Diosmetin	5,7,3'	4'	-	48.63 ± 0.97 ^hj^
Norwogonin	5,7,8	-	-	68.25 ± 1.50 ^k^
Oroxylin A	5,7	6	-	82.86 ± 1.74 ^mo^
Luteolin	5,7,3',4'	-	-	82.97 ± 1.91 ^mo^
Baicalein	5,6,7	-	-	100
Scutellarein	5,6,7,4'	-	-	100
6-Hydroxyluteolin	5,6,7,3',4'	-	-	100
*Flavon-3-ols*				
3-Hydroxyflavone	3	-	-	10.31 ± 0.24 ^ac^
Galangin	3,5,7	-	-	22.48 ± 0.54 ^e^
Spiraeoside	3,5,7,3',4'	-	4'	30.49 ± 0.73 ^g^
Astragalin	3,5,7,3'	-	3	32.76 ± 0.78 ^h^
Isoquercitrin	3,5,7,3',4'	-	3	57.73 ± 1.33 ^j^
Kaempferol	3,5,7,4'	-	-	57.76 ± 1.38 ^i^
Isorhamnetin	3,5,7,4'	3'	-	59.67 ± 1.47 ^jk^
Datiscetin	3,5,7,2'	-	-	61.38 ± 1.65 ^jk^
Morin	3,5,7,2',4'	-	-	69.65 ± 1.67 ^jl^
Tamarixetin	3,5,7,3'	4'	-	71.38 ± 1.71 ^km^
Rhamnetin	3,5,3',4'	7	-	73.41 ± 1.83 ^l^
Isomyricitrin	3,5,7,3',4',5'	-	3	76.63 ± 1.84 ^m^
Fisetin	3,7,3',4'	-	-	82.34 ± 1.97 ^n^
Azaleatin	3,7,3',4'	5	-	83.10 ± 1.98 ^mo^
Patuletin	3,5,7,3',4'	6	-	88.50 ± 2.30 ^o^
Quercetin	3,5,7,3',4'	-	-	95.50 ± 2.48 ^p^
Myricetin	3,5,7,3',4',5'	-	-	100
Quercetagetin	3,5,6,7,3',4'	-	-	100

All values correspond to mean values ± standard deviation of three replicates. Values with different letters (a–p) indicate statistically significant differences among groups at *p* < 0.05 by one-way ANOVA.

During the transition from chrysin (5,7-OH) to apigenin (5,7,4'-OH), a slight rise in activity is observed (from 11.87% to 18.01%). The significant influence of the hydroxyl at the C-4' position proves the fact that its substitution (as in acacetin) decreases the activity of the compound (10.23%), while a genkwanin having a methoxy group at C-7 is similar to apigenin (19.02%). 

A further increase in the amount of hydroxyl groups in the B-ring leads to a sharp increase in activity, for example, the activity of luteolin (5,7,3',4'-OH) amounts to 82.97%. Interestingly, the effect of the hydroxyl groups in positions C-3' and C-4' on chelating activity of the compounds manifests in different ways: the replacement of the hydroxyl at C-4' (diosmetin) decreases the efficiency to 48.63%, but in the case of 3'-methoxy-luteolin (chrysoeriol), the activity falls by more than fourfold.

Flavone derivatives are characterized by the presence of a hydroxyl group at the C-3 position. The maximum intensity of the activity was found for the compounds containing three ordinary hydroxyls in the A-ring, *i.e.*, quercetagetin (3,5,6,7,3',4'-OH), and in the B-ring, *i.e.*, myricetin (3,5,7,3',4',5'-OH). Basic patterns identified for flavone derivatives are typical for derivatives of flavon-3-ol.

The presence of hydroxyl at C-3 has a positive impact on the effectiveness of chelation of Fe^2+^ ions. This fact is demonstrated by comparative analysis of two series of flavone and flavone-3-ol derivatives:


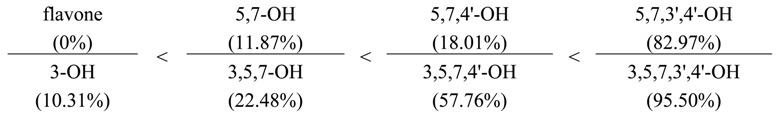


The insertion of the hydroxyl group in the C-3 position of flavone results in chelating activity of the compound, which is due to the possibility of formation of a complex between the Fe^2+^ atom, the hydroxyl at C-3 and the oxygen of the carbonyl group at C-4 [21]. In the case of substitution of the hydroxyl at C-3, for example, by the residues of carbohydrates, the efficiency of the chelation of the compounds is reduced. Thus, the activity of kaempferol, quercetin and myricetin in comparison with their 3-O-glucosides was significantly higher:


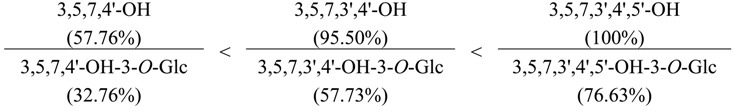


The substitution of the hydroxyl at C-5 has little effect on the activity of the compounds. For example, the efficiency of azaleatin (5-methoxyquercetin) was 83.10% and was lower than that of quercetin (95.50%), whereas for fisetin (de-hydroxylated analogue of quercetin), it was 82.34%.

The importance of the hydroxyl group in the C-3' position in the processes of chelation previously identified for flavones was confirmed by the analysis of the flavone-3-ols. The activity of 4'-methoxy-quercetin (tamarixetin) was similar to that of 7-methoxyquercetin (rhamnetin) and exceeded the activity of the 3'-methoxy-quercetin (isorhamnetin) (71.38%, 73.41% and 59.67%, respectively).

It should be noted that for the 2'-hydroxy analogue of kaempferol, *i.e.*, morin (3,5,7,2',4'-OH), relatively high activity was observed (69.65% *vs.* 57.76% for kaempferol), close to that of tamarixetin (3,5,7,3'-OH-4'-MeO; 71.38%). Moreover, datiscetin (3,5,7,2'-OH), which does not contain hydroxyl at C-4', also had a high capacity for chelating Fe^2+^ ions (61.38%). These findings suggest an important role of the 2'-hydroxy-substituted flavonoids in metal chelation.

The revealed patterns of influence of the structure of flavonoids on the effectiveness of the chelation of Fe^2+^ ions are confirmed by the facts established earlier as a result of numerous studies by various research groups [19,23,26,27,28,29,30,31,32,33,34]. In particular, among the major structural features that provide the primary influence on the ability of compounds to bind metal ions are the following:

1. the 2,3-double bond in conjugation with 4-keto-function;
2. the 3-hydroxy-4-keto- or 5-hydroxy-4-keto-functions;
3. the 5,7-di-hydroxy- and additionally, 3-hydroxy-functions (5,7-di-OH; 3,5,7-tri-OH);
4. the *o*-dihydroxyl (catechol) structure of the B-ring (3',4'-di-OH) and/or the A-ring (7,8-di-OH);
5. the galloyl structure of the B-ring (3',4',5'-tri-OH) and/or the A-ring (5,6,7-tri-OH).

All of the rules mentioned above are confirmed in the results of the present study. However, it should be noted that some issues concerning the structural features that have a positive influence on the process of chelation should be explored in more depth through additional studies. 

In particular, the presence of 3,2'-dihydroxy function considered as an additional structural feature results in increased Fe-CA. One of the representatives of the compounds of this feature is morin (3,5,7,2',4'-pentahydroxyflavone), a known metal ion chelating agent. According to early data, morin can form complexes with bivalent metals such that the morin:metal ratio is equal to 2:1 [35]. The detection of high chelating activity of datiscetin, which is a 4'-dehydroxylated analogue of morin, indicates the possibility of formation of complexes of the same type. This group is also found in the structure of other flavonoids, including datin (3,5,2'-trihydroxy-7-methoxyflavone), viscidulin I (3,5,7,2',6'-pentahydroxyflavone) and 5'-hydroxymorin [24]. Additional data is necessary to explain why the substitution or deletion of a hydroxyl group at the C-3' position causes a significant reduction in the activity of the chelating compound. 

### 2.3. Characterization of Fe-CA of Flavonoid Mixtures by HPLC Using a Prechromatographic Reaction with Fe^2+^ Ions

**Figure 3:**
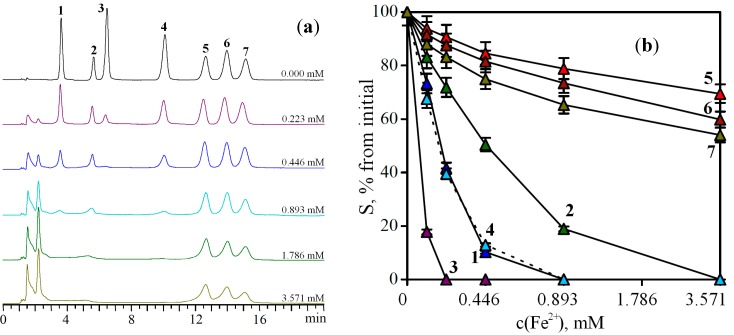
(**a**) Chromatograms of flavonoid mixture before (0.000 mM) and after prechromatographic reaction with Fe^2+^ ions (0.223–3.571 mM). (**b**) Reduction of peak areas (S) of flavonoids in the mixture after prechromatographic reaction with different concentration of Fe^2+^ ions.

Given the fact that plants are a complex matrix containing different classes of compounds with diverse structural features, it is of interest to check the applicability of the developed approaches to the analysis of mixtures of flavonoids. An artificial mixture of flavonoids containing compounds with different levels of chelating action was composed. The mixture included components with high (baicalein), medium (quercetin, kaempferol, oroxylin A) and low binding activity (eupatorin, acacetin, galangin). Each sample contained an equal amount of flavonoids (0.3 mM) but had different contents of Fe^2+^ (Figure 3).

With increasing concentration of Fe^2+^ ions in the sample, a different rate of reduction of the areas of the chromatographic peaks for compounds with diverse effectiveness of chelation was observed. For example, at the concentration of 0.223 mM Fe^2+^ a pronounced decrease in the peak area of baicalein was typical, and at a concentration of 0.446 mM Fe^2+^, a complete disappearance of the peak of the compound was observed. A similar trend was found for quercetin and oroxylin A when the iron concentration in the sample was 1.786 mM. The disappearance of the kaempferol peak occurred at the Fe^2+^ ions concentration of 3.571 mM. The maximum fall of the peak areas of inactive compounds (eupatorin, acacetin, galangin) was only 20%–40% of the baseline, even in solutions with a high content of Fe^2+^ ions.

1. quercetin; 2. kaempferol; 3. baicalein; 4. oroxylin A; 5. eupatorin; 6. acacetin; 7. galangin.

Presenting the data as dependence “concentration of Fe^2+^ ions—the area of the chromatographic peak” for different compounds, one can notice the presence of a dose-dependent character of the chelation of Fe^2+^ ions. Moreover, the effectiveness of the chelation of Fe^2+^ ions by a compound is probably a function of the rate of the chelate-forming reaction between the compound and available chelating Fe^2+^ ions. For example, baicalein is an effective chelator because of structural features that allow it to bind Fe^2+^ ions with higher speed, unlike eupatorin, which is also capable of binding Fe^2+^ ions, but at a slower rate. 

Thus, these studies have shown that the HPLC method is a useful tool for detection of compounds that are able to chelate Fe^2+^ ions. This method allows identifying potential chelators using a two-step procedure, namely, the chromatographic profiling of the sample at the first stage, and the analysing the sample after prechromatographic reaction with Fe^2+^ ions at the second stage.

### 2.4. Characterization of Fe-CA of Scutellaria baicalensis by HPLC and Spectrophotometry

To demonstrate the capabilities of the developed method, we have attempted to study the Fe-CA of a real plant, *Scutellaria baicalensis* Georgi. (Lamiaceae family). This plant species contains compounds with high binding activity [32,36]. In spite of the known facts about the chelating properties of the individual flavonoids of *S. baicalensis*, there is currently no scientific information about the comparative activity of the various organs of this species. This information allows evaluation of the prospect of application of various parts of the plant as a source of biologically active compounds. The information available concerns mainly the use of underground parts and information about the application of the aerial part, but is limited to a small number of works.

Chemical analysis of the different morphological groups (organs) of *S. baicalensis* is necessary for the first stage of the investigation. Chromatographic analysis of the extracts from the flowers, stems, leaves and roots of *S. baicalensis* was performed. It was revealed that flavonoids in *S. baicalensis* distribute unevenly, *i.e.*, each part has a characteristic flavonoid profile (Table 2).

**Table 2:** Content of phenolic compounds in organs and extracts of *S. baicalensis* (mg·g^−1^ of dry plant/extract weight).

Compound	Flowers			Leaves			Stems		Roots	
	Buds	Beginning of flowering	Mass flowering	Young leaves of the main stem	Mature leaves of the main stem	Leaves of the lateral stem	Main stems	Lateral stems	Main root	Adventi-tious root
*Organs*										
A7G	14.10	21.57	21.30	2.23	2.21	2.54	0.52	1.70	tr.	tr.
B	tr.	tr.	tr.	tr.	tr.	tr.	tr.	tr.	4.44	20.50
B7G	26.06	7.26	4.89	3.80	4.11	4.52	0.17	0.43	195.57	105.41
C	tr.	tr.	tr.	tr.	tr.	tr.	n.d.	n.d.	0.42	1.10
C7G	67.37	45.11	31.61	37.55	23.38	44.61	0.26	1.22	n.d.	n.d.
CC1	n.d.	n.d.	n.d.	n.d.	n.d.	n.d.	n.d.	n.d.	7.50	5.84
CC2	n.d.	n.d.	n.d.	n.d.	n.d.	n.d.	n.d.	n.d.	5.26	3.64
DB7G	tr.	tr.	0.48	0.23	0.14	0.24	tr.	tr.	2.62	1.54
DS7G	tr.	tr.	tr.	40.21	69.21	66.18	6.86	20.14	tr.	tr.
Fl7G	n.d.	n.d.	n.d.	n.d.	n.d.	n.d.	n.d.	n.d.	4.17	2.26
IS7G	0.75	tr.	n.d.	0.65	0.28	0.69	0.25	0.41	tr.	tr.
L7G	23.49	12.17	10.28	tr.	tr.	tr.	tr.	tr.	tr.	tr.
N7G	4.84	0.75	0.61	0.28	0.17	0.32	0.14	0.12	5.54	5.69
O7G	n.d.	n.d.	n.d.	n.d.	n.d.	n.d.	n.d.	n.d.	7.93	3.74
S7G	14.73	2.43	2.32	3.77	2.82	5.43	2.27	5.61	tr.	tr.
W	n.d.	n.d.	n.d.	n.d.	n.d.	n.d.	n.d.	n.d.	0.48	2.29
W7G	n.d.	n.d.	n.d.	n.d.	n.d.	n.d.	n.d.	n.d.	41.59	26.47
*Extracts*										
A7G	23.94	38.48	40.59	7.43	6.02	9.72	2.38	7.84	1.12	tr.
B	1.04	0.93	0.60	0.53	0.47	0.86	0.15	0.39	10.45	41.62
B7G	45.24	11.39	9.08	12.68	14.52	16.20	0.81	2.43	460.16	214.98
C	4.16	2.18	1.12	1.16	1.12	0.83	n.d.	n.d.	1.04	2.29
C7G	117.86	82.14	63.28	127.03	75.82	150.32	1.37	5.63	n.d.	n.d.
CC1	n.d.	n.d.	n.d.	n.d.	n.d.	n.d.	n.d.	n.d.	17.69	12.82
CC2	n.d.	n.d.	n.d.	n.d.	n.d.	n.d.	n.d.	n.d.	11.38	7.42
DB7G	1.83	1.86	0.84	0.82	0.52	0.81	tr.	tr.	6.53	3.10
DS7G	2.10	1.12	0.57	135.30	223.96	221.48	32.84	92.93	0.64	0.39
Fl7G	n.d.	n.d.	n.d.	n.d.	n.d.	n.d.	n.d.	n.d.	10.48	5.06
IS7G	1.29	0.83	n.d.	1.89	0.73	2.32	1.19	1.58	1.86	0.93
L7G	40.78	20.66	20.69	0.32	0.16	1.78	tr.	0.24	2.09	0.52
N7G	8.23	1.44	1.17	1.03	0.53	1.73	0.63	0.60	14.89	10.02
O7G	n.d.	n.d.	n.d.	n.d.	n.d.	n.d.	n.d.	n.d.	19.96	7.83
S7G	24.09	4.38	4.45	14.07	9.04	18.29	10.63	26.43	0.96	1.12
W	n.d.	n.d.	n.d.	n.d.	n.d.	n.d.	n.d.	n.d.	1.10	4.65
W7G	n.d.	n.d.	n.d.	n.d.	n.d.	n.d.	n.d.	n.d.	99.60	53.78

A7G. apigenin-7-*O*-glucuronide; B. baicalein; B7G. baicalin; C. chrysin; C7G. chrysin-7-*O*-glucuronide; CC1. chrysin-6-*C*-arabinoside-8-*C*-glucoside; CC2. chrysin-6-*C*-glucoside-8-*C*-arabinoside; DB7G. dihydrobaicalin; DS7G. dihydroscutellarin; Fl7G. 5,7,8-trihydroxy-6-methoxyflavone-7-*O*-glucuronide; IS7G. isoscutellarin; L7G. luteolin-7-*O*-glucuronide; N7G. norwogonoside; O7G. oroxyloside A; S7G. scutellarin; W. wogonin; W7G. wogonoside. tr.—traces (<limit of quantification); n.d.—not detected (<limit of detection).

The ability of flowers to accumulate chrysin-7-*O*-glucuronide and luteolin-7-*O*-glucuronide was marked in all periods of growth (from buds to the phase of mass flowering). It should be noted that the content of these compounds was reduced in flowers during their growth, as was the total flavonoid content. High concentrations of baicalin, scutellarin and norwogonoside were typical for buds. The concentration of apigenin-7-*O*-glucuronide was higher in adult flowers than in the buds. The leaves of *S. baicalensis* are used for storage of dihydroscutellarin and chrysin-7-*O*-glucuronide, and the concentrations of which are higher in mature leaves. Apigenin-7-*O*-glucuronide, baicalin, norwogonoside and scutellarin are minor compounds of this organ. The predominant flavonoid of *S. baicalensis* stems is dihydroscutellarin, the highest concentration of which is in the lateral stems. In general, the composition of flavonoids of stems is close to that of the leaves, except for the minor content of chrysin-7-*O*-glucuronide. The roots have a special chemical feature consisting of a high concentration of baicalin and wogonoside, which has previously has been shown in many studies. However, a high concentration of aglycone of baicalin (baicalein) was observed for adventitious roots (*ca*. fivefold more than in the main root).

Based on the identified features, a variety of Fe-CA levels in extracts from different organs is expected. Extracts were obtained from all the above-listed parts and investigated for their Fe-CA. Chromatographic analysis showed that the composition of the extracts is similar to that of producing parts of them (Table 2).

**Figure 4:**
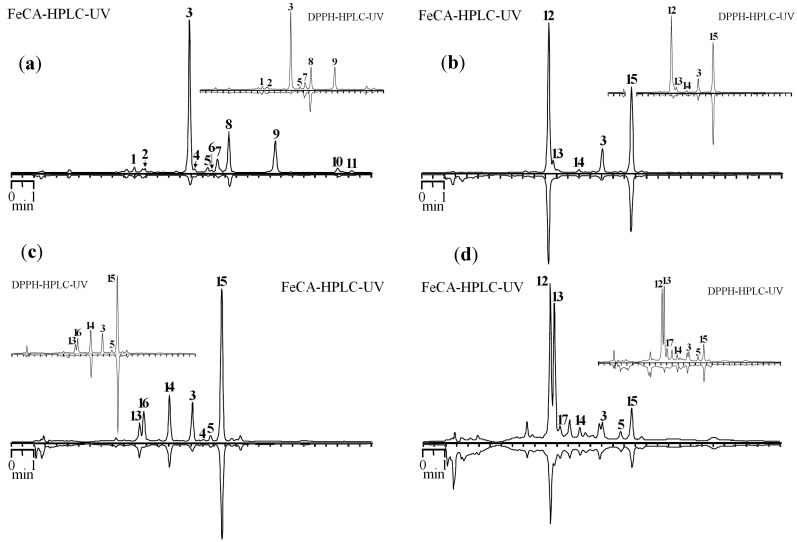
HPLC chromatograms of *S. baicalensis* extracts before (top chromatogram) and after prechromatographic reaction with Fe^2+^ ions (bottom chromatogram) (FeCA-HPLC-UV). Extracts: (**a**) main roots; (**b**) mature leaves of main stem; (**c**) flowers, mass flowering period; (**d**) main stem. On cuts—HPLC chromatograms of the same extracts before (top chromatogram) and after prechromatographic reaction with DPPH^•^ radicals (bottom chromatogram) (DPPH-HPLC-UV).

The developed method was applied to study Fe-CA of extracts obtained from various parts of *S. baicalensis*. Some chromatograms of roots, leaves, flowers and stems of *S. baicalensis* are presented in Figure 4. 

1. chrysin-6-*C*-arabinoside-8-*C*-glucoside; 2. chrysin-6-*C*-glucoside-8-*C*-arabinoside; 3. baicalin; 4. dihydrobaicalin; 5. norwogonoside; 6. 5,7,8-trihydroxy-6-methoxyflavone-7-*O*-glucuronide; 7. oroxyloside A; 8. wogonoside; 9. baicalein; 10. wogonin; 11. chrysin; 12. dihydroscutellarin; 13. scutellarin; 14. apigenin-7-*O*-glucuronide; 15. chrysin-7-*O*-glucuronide; 16. luteolin-7-*O*-glucuronide; 17. isoscutellarin.

The largest decrease in the peak area was observed for baicalin, baicalein and wogonoside in the roots. It should be noted that minor 5,7,8-trihydroxy-6-methoxyflavone-7-*O*-glucuronide, oroxyloside A and norwogonoside may be characterized as good chelators. Dihydroscutellarin and chrysin-7-*O*-glucuronide, predominant compounds of the flower extract, have a weak Fe-CA, while scutellarin and baicalin are main chelators of this extract.

The dominance of chrysin-7-*O*-glucuronide, which exhibits weak chelating properties, is also observed in the extract from the leaves. Luteolin-7-*O*-glucuronide and baicalin are compounds that are responsible for the existence of Fe-CA. Dihydroscutellarin and scutellarin are the main constituents of the extract from the stems. However, Fe-CA of the former compound is significantly lower than that of the latter, the presence of which determines the existence of the effect in the extract from this organ.

Presentation of the results in the form of coupled chromatograms, one of which is inverted, allows initial evaluation in a more convenient form. For comparison of the capabilities of chromatographic techniques using prechromatographic treatment of the sample, we conducted an investigation by applying the DPPH-HPLC method. Despite the proximity of the structural features determining the presence of antiradical action with those for Fe^2+^-chelating activity, similar chromatographic profiles for DPPH-HPLC and FeCA-HPLC methods would be expected, but sometimes profiles may differ. In particular, a significant decrease in the peak area of dihydroscutellarin after the treatment of the extract from the flowers by the DPPH radical is observed. The presence of a single peak (chrysin-7-glucuronide) in the inverted chromatogram is the result of this. However, this compound showed weak Fe^2+^-chelating properties that, in the case of FeCA-HPLC, resulted in two peaks on Fe^2+^-treated chromatogram. A similar phenomenon was observed for the extract from the stems, whose also contain a significant amount of dihydroscutellarin. The main reason of these differences is the presence of 2,3-saturated bond in dihydroscutellarin resulted in high antiradical action but pronounced reduction of Fe^2+^-chelating properties [34]. Thus, the simultaneous use of DPPH-HPLC and FeCA-HPLC for characterization of plants (extracts) obtains a more complete picture of the active compounds and features of their antioxidant action.

In order to quantify the degree of Fe-CA in extracts from the parts of *S. baicalensis*, we defined the indicators of Fe-CA using a spectrophotometric method. It was found that the values of Fe-CA ranged from 10.09 μM Fe^2+^ g^−1^ for flowers in a phase of mass flowering to 471.70 μM Fe^2+^ g^−1^ for the main root (Table 3). 

**Table 3:** Fe^2+^-Chelating activity (Fe-CA), reduction of total peaks areas (Δ*S^t^*) and chemical parameters of extracts from different *S. baicalensis* organs.

Sample	Fe-CA, μM Fe^2+^ g^−1^ *^,^**	Δ*S^t^*, %	Total flavonoid content, mg·g^−1^ **	“Active” flavonoid content, mg·g^−1^ **
Flowers				
Buds	102.63 ± 2.36 ^f^	48.07 ± 1.11 ^j^	270.56	119.38
beginning of flowering	33.29 ± 0.76 ^de^	35.79 ± 0.79 ^hi^	165.41	38.80
mass flowering	30.02 ± 0.69 ^de^	33.22 ± 0.70 ^h^	142.39	35.99
Leaves				
young leaves of the main stem	20.92 ± 0.48 ^c^	27.46 ± 0.63 ^g^	302.26	28.63
mature leaves of the main stem	18.64 ± 0.41 ^bc^	22.86 ± 0.57 ^ig^	332.89	24.72
leaves of the lateral stem	32.67 ± 0.75 ^de^	34.37 ± 0.79 ^h^	424.34	38.86
Stems				
main stem	10.14 ± 0.24 ^a^	18.20 ± 0.43 ^i^	50.00	12.22
lateral stems	27.16 ± 0.59 ^d^	30.42 ± 0.69 ^gh^	138.07	30.09
Roots				
main root	471.70 ± 12.26 ^h^	85.57 ± 1.97 ^l^	659.95	518.99
adventitious root	253.08 ± 6.07 ^g^	59.19 ± 1.30 ^k^	366.53	281.15

* Average of three analyses (±SD). All values correspond to mean values ± standard deviation of three replicates. Values with different letters (a–k) indicate statistically significant differences among groups at *p* < 0.05 by one-way ANOVA; ** of dry extract weight.

Flowers, except buds, were characterized by a relatively low Fe-CA and intermediate rates were revealed for the extract from leaves, while the extract from the roots was the most active. The different flavonoid content in these parts is the reason for this phenomenon, and it should be noted that various flavonoids found in *S. baicalensis* possess different Fe^2+^-chelating activities, so baicalein, baicalin, 5,7,8-trihydroxy-6-methoxyflavone-7-*O*-glucuronide, luteolin-7-*O*-glucuronide, norwogonoside, oroxyloside A and scutellarin can be referred to as “active” flavonoids. The remaining compounds are characterized as weak chelators.

**Figure 5:**
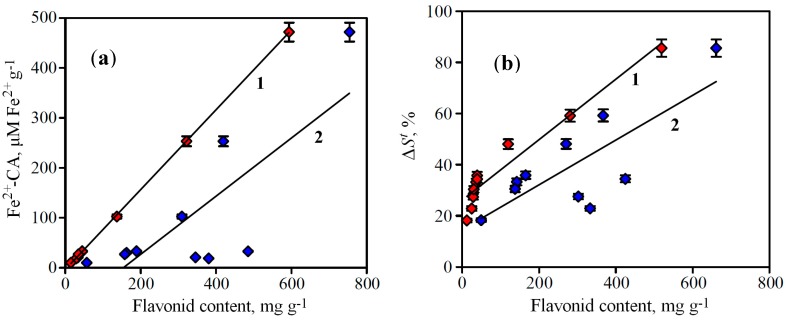
(**a**) Correlations between flavonoid content in *S. baicalensis* extracts and their Fe-CA determined by spectrophotometry (Fe^2+^-CA). (**b**) Correlations between flavonoid content in *S. baicalensis* extracts and their Fe-CA determined by FeCA-HPLC (Δ*S^t^*).

Therefore, to describe the extracts, we used two measures: total flavonoid content and “active” flavonoid content. The value of total flavonoid content is calculated as a sum of all identified flavonoid compounds (HPLC) and “active” flavonoid content is a sum of most active compound, including baicalein, baicalin, 5,7,8-trihydroxy-6-methoxyflavone-7-*O*-glucuronide, luteolin-7-*O*-glucuronide, norwogonoside, oroxyloside A and scutellarin. The use of regression analysis revealed the presence of linear correlations between flavonoid content in *S. baicalensis* extracts and their Fe-CA (Figure 5a). The value of the regression coefficients indicates that the rates of flavonoid content and Fe-CA depend on each other, *i.e.*, while the flavonoid content is high, the iron-chelating activity is also high.

1. “active” flavonoid content, 2. total flavonoid content. (a) *r*^2^ = 0.9999 for “active” flavonoid content and *r*^2^ = 0.6167 for total flavonoid content. (b) *r*^2^ = 0.9329 for “active” flavonoid content and *r*^2^ = 0.6005 for total flavonoid content.

However, the dispersion of *r*^2^-values for relationship “active” flavonoid content—Fe-CA is much smaller than for total flavonoid content—Fe-CA one. This phenomenon confirms the predominant role of active flavonoids in availability of iron-chelating activity, so the use of this criterion for the correlation investigations is more informative. 

To compare the efficiency of the chromatographic method of determination of Fe^2+^-chelating activity to that of the traditionally used spectrophotometric method, we investigated the presence of correlations between the results of both methods. Parameter reduction of total peaks areas (Δ*S^t^*) was proposed as a quantitative criterion for assessing Fe^2+^-chelating activity by the FeCA-HPLC method. This parameter is the ratio of the sum of areas of all chromatographic peaks after treatment of the sample to that before treatment (in per cent):

$$\Delta S^{t},\%=100\%-(\frac{S^{after}}{S^{before}}\cdot 100\%)$$

where *S^after^* is the total area of all chromatographic peaks after Fe^2+^ treatment, and *S^bef^°^re^* is the total area of all chromatographic peaks before Fe^2+^ treatment. The calculation of this parameter for the *S. baicalensis* extracts showed that the rate varied from 18.20% (extract from the stems, the least active sample) to 85.57% (extract from the roots, the most active sample) (Table 3). This parameter is in good correlation with both indices of flavonoid content in extracts, “active” flavonoid content and total flavonoid content. As in the case of Fe-CA determined by spectrophotometry the dispersion of *r*^2^-values in for relationship “active” flavonoid content—Δ*S^t^* is much smaller than for total flavonoid content—Δ*S^t^* (Figure 5b). 

**Figure 6:**
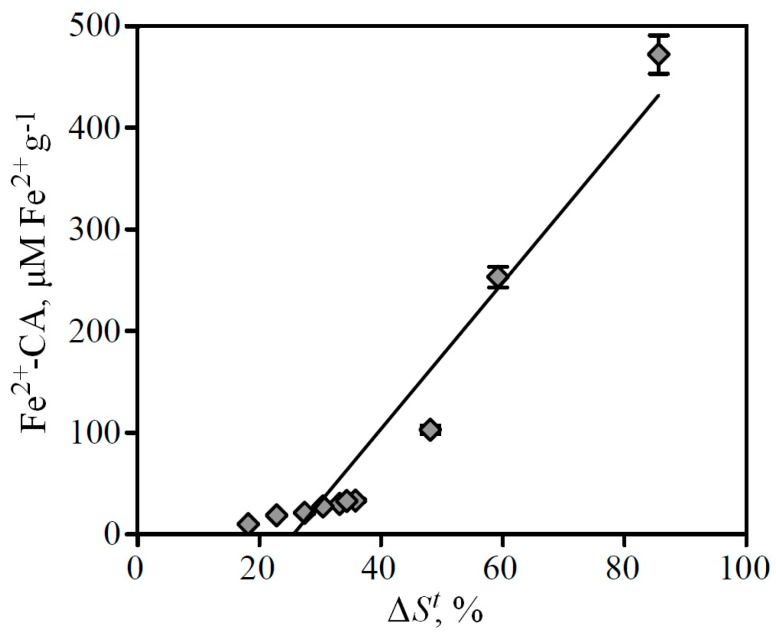
Correlation between values of Fe-CA determined by FeCA-HPLC (Δ*S^t^*) and spectrophotometry (Fe^2+^-CA).

Comparative analysis of the two methods' data showed that the correlation between values of reduction of total peaks areas (Δ*S^t^*) in FeCA-HPLC and Fe-CA index in spectrophotometry is characterized by a high correlation coefficient (*r*^2^ = 0.9307), which indicates a strong bond of both parameters (Figure 6).

Our studies have shown the possibility of combining the advantages of the HPLC method and prechromatographic derivatization of samples by Fe^2+^ ions for investigation of Fe^2+^-chelating activity of natural compounds. The economic feasibility of this method is obvious because it does not require additional chemicals and is rapid. This method is sensitive because it uses an HPLC technique for detection of quantitative and qualitative changes in the sample after Fe^2+^ treatment. Undoubtedly, it is not possible to transfer the results of this method to processes in the human organism. However, obtained results may give an idea of the Fe^2+^ chelating efficacy of different plant metabolites. 

## 3. Experimental Section

### 3.1. Reference Compounds

Reference compounds with purity greater than 96% were used. This included commercially available compounds of the *flavone series*: flavone, chrysin, apigenin, wogonin, norwogonin, oroxylin A, 6-hydroxyluteolin, luteolin, baicalein, scutellarein from Sigma-Aldrich (St. Louis, MO, USA); dimethoxychrysin, sinensetin, tectochrysin, acacetin, chrysoeriol, genkwanin, eupatorin, diosmetin from Extrasynthese (Lyon, France); compounds of the *flavon-3-ol series*: 3-hydroxyflavone, kaempferol, isorhamnetin, morin, rhamnetin, fisetin, azaleatin, quercetin, myricetin, isoquercitrin, isomyricitrin from Sigma-Aldrich; galangin, datiscetin, tamarixetin, patuletin, quercetagetin, spiraeoside, astragalin from Extrasynthese. Apigenin-7-*O*-glucuronide, baicalin, chrysin-7-*O*-glucuronide, chrysin-6-*C*-arabinoside-8-*C*-glucoside, chrysin-6-*C*-glucoside-8-*C*-arabinoside, dihydro-baicalin, dihydroscutellarin, 5,7,8-trihydroxy-6-methoxyflavone-7-*O*-glucuronide, isoscutellarin, luteolin-7-*O*-glucuronide, norwogonoside, oroxyloside A, wogonoside were isolated previously from *Scutellaria baicalensis* and *Scutellaria scordiifolia* [36,37,38,39,40].

### 3.2. Plant Material

Plants of *Scutellaria baicalensis* Georgi grown in hydroponic culture were used for analysis and extracts preparation. *S. baicalensis* seeds of natural population (Dog’e village, Zabaikal’skii Krai, Russia) germinated in sand (with 4–5 first true leaves), then plants were transplanted to Hoagland solution [41]. Uniform plants were cultivated in a plastic 45-L Drip Hydroponic System GH Aqua Farm (Fleurance, France) with continual aeration of the solution in Grow Tents Secret Jardin Dark Street 150 v. 2.50 (Genval, Belgium). The cultivation process was under controlled conditions: 12-h photoperiod (High Pressure Sodium Lamp, HO Lucalox 600 W, General Electric, Budapest, Hungary) with a 25/20 °C day/night temperature and a relative humidity of 50% (Microprocessor-based Control Unit Dzagi Grow, Perm, Russia). Solutions were renewed weekly to prevent nutrient depletion. Plants were cultivated in hydroponics until the phase of full flowering (three months), then they were pulled out, washed, divided in different organs and dried *in vacuo* at 40 °C. For analytical HPLC, total probes of the sample from 15–20 specimens of *S. baicalensis* were used. For extract preparation, total probes of the sample from 100–250 specimens of *S. baicalensis* were used.

### 3.3. Preparation of S. baicalensis Extracts 

*S. baicalensis* sample was powdered in a mechanical grinder. The powdered sample was weighted accurately (50 g), and extracted twice with 70% ethanol (1 L) in an ultrasonic bath for 40 min at 45 °C. The extracted solutions were filtered through a cellulose filter and evaporated *in vacuo* until dryness using a rotary evaporator. The extracts yields were (w/w) 58.1% for flowers buds, 54.2% for flowers at the beginning of flowering period, 52.5% for flowers at the mass flowering period, 30.0% for young leaves of the main stem, 31.3% for mature leaves of the main stem, 26.7% for leaves of the lateral stem, 21.3% for main stems, 25.8% for lateral stems, 59.4% for main roots and 50.0% for adventitious roots.

### 3.4. Extraction and Sample Preparation for HPLC Analysis

Reference samples of flavonoids were used as solutions in DMSO (Fe^2+^-chelating activity experiments of individual compounds or mixtures) or methanol (analytical HPLC of *S. baicalensis* samples) in 1–10 mg/mL concentration, stored at −20 °C before analysis. The dried and powdered *S. baicalensis* plant samples (200 mg) were extracted with 70% ethanol (5 mL) in an ultrasonic bath for 40 min at 45 °C. The extracted solutions were filtered through a 0.22-μm PTFE syringe filter before injection into the HPLC system for analysis. *S. baicalensis* extract samples (10 mg) were dissolved in 70% ethanol (1 mL) in an ultrasonic bath for 10 min at 45 °C. The extracted solutions were filtered through a 0.22-μm PTFE syringe filter before injection into the HPLC system for analysis. 

### 3.5. Microcolumn HPLC-UV

All HPLC experiments were performed on a microcolumn chromatograph Econova MiLiChrom A-02 (Novosibirsk, Russia) coupled with UV-detector, using ProntoSIL-120-5-C18 AQ column (2 × 75 mm, 5 μm; Metrohm AG; Herisau, Switzerland), column temperature was 35 °C. Mobile phase A was 0.2 М LiClO_4_ in 0.006 M HClO_4_ and mobile phase B was acetonitrile. The injection volume was 1 μL, and elution was at 150 μL/min. Gradient programmes: *flavonoid aglycones*—0–2 min, 5%–10% B; 2–8 min, 10%–100% B; 8–10 min, 100% B, 10–12 min, 100%–5% B; *flavonoid glycoside—*0–5 min, 11%–18% B; 5–9 min, 18% B; 9–10 min, 18%–20% B, 10–12 min, 20%–25% B, 12–16 min, 25% B, 16–20 min, 25%–100% B, 20–24 min, 100% B, 24–26 min, 100%–11% B; *S. baicalensis extracts*—0–16 min, 15%–60% B; 16–20 min, 60%–15% B. Detector wavelength was 270 nm. 

### 3.6. Fe^2+^-Chelating Activity HPLC-UV Procedure

Briefly, 100 μL of sample solution was added to 100 μL of FeSO_4_∙7H_2_O solution (125 μg/mL) in PBS (pH 6.0). The mixture was shaken for a few seconds and then incubated at 37 °C for 30 min. Then the sample was filtered through a 0.22-μm membrane filter and analysed using the above-mentioned HPLC conditions. The untreated sample was prepared by adding 100 μL of sample solution and 100 μL of PBS (pH 6.0). Initial sample solution concentrations were 1 mg/mL for reference samples of flavonoids and 10 mg/mL for *S. baicalensis* extracts. 

### 3.7. Residual Concentration of Fe^2+^ Ions

The filtrate after prechromatographic reaction of sample solution with Fe^2+^ ions (*3.6*) was used for determination of the residual concentration of Fe^2+^ ions. Aliquot (10 μL) of the filtrate, 10 μL of hydroxylamine hydrochloride solution (0.1 mg/mL) in water and 20 μL of sodium hydroxide solution (0.04 mg/mL) in water were transferred to a 96-well plate. The plate was incubated at 37 °C with continuous shaking (800 revolutions per minute) for 10 min. Then, 150 μL of *O*-phenanthroline solution (5 mg/mL) in MeOH and 100 μL of deionised water were added and the plate was shaken vigorously (10 min). Absorbance of the sample solution was measured at 500 nm using a microplate spectrophotometer, Uniplan (Moscow, Russia). The value of the residual concentration of Fe^2+^ ions was calculated using graph of absorbance (*A*^500^) of working solutions against Fe^2+^ concentration (mM).

### 3.8. Fe^2+^-Chelating Activity Spectrophotometric Procedure

The Fe^2+^-chelating activity of extracts was determined by the *o*-phenanthroline method [42]. Aliquots (10–100 μL) of the sample solution (50 mg/mL) and 25 μL of FeSO_4_∙7H_2_O solution (22.24 mg/mL) in PBS (pH 6.0) were transferred to a 96-well plate. Final volumes of probe (125 μL) were corrected by adding PBS (pH 6.0). The blank solution contained 100 μL of PBS (pH 6.0) and 25 μL of FeSO_4_∙7H_2_O solution (22.24 mg/mL) in PSB (pH 6.0). The plate was incubated at 37°C with continuous shaking (800 revolutions per minute) for 40 min. Then, 225 μL of MeOH was added and the plate was shaken vigorously (1 min) and centrifuged (6000 *g*, 10 min). One hundred μL of supernatant was mixed with 200 μL of *O*-phenanthroline solution (20 mg/mL) in MeOH. After 20 min, absorbance of the sample solutions was measured at 500 nm using a microplate spectrophotometer, Uniplan (Moscow, Russia). Lower absorbance of the reaction mixture indicated higher Fe^2+^-chelating activity, which was analysed from the graph (inhibition percentage plotted against concentration of substance). The effectiveness of Fe^2+^-chelating activity was measured as μM of Fe^2+^ ions chelated by 1 g of extract (μM Fe^2+^ g^−1^). 

### 3.9. DPPH-HPLC-UV Procedure

Briefly, 100 μL of *S. baicalensis* extract solution in 70% ethanol (25 mg/mL) was added to 100 μL DPPH^•^ radical solution in methanol (20 mg/mL). The mixture was shaken for a few seconds and left to stand in the dark for 30 min at room temperature. Then the sample was filtered through a 0.22-μm membrane filter. The untreated sample was prepared by adding 100 μL of *S. baicalensis* extract solution in 70% ethanol (25 mg/mL) to 100 μL of methanol. HPLC analysis was performed using the above-mentioned conditions.

### 3.10. Statistical Analysis

Statistical analyses were performed using a one-way analysis of variance (ANOVA), and the significance of the mean difference was determined by Duncan’s multiple range test. Differences at *p* < 0.05 were considered statistically significant. The results are presented as mean values ± SD (standard deviations) of the three replicates.

## 4. Conclusions 

A new HPLC-assisted method (FeCA-HPLC) for investigation of Fe^2+^-chelating activity was developed. This method is suitable for laboratory estimation of main compounds caused the chelating activity of plant extracts. Using FeCA-HPLC can significantly accelerate the process of determining the active metabolites. The described structural features causing the activity of compounds are present not only in flavones and flavonols but also in other classes of natural substances, *i.e.*, catechins, isoflavonoids, phenylpropanoids, tannins, coumarins, whose may be analysed by FeCA-HPLC. Good agreement between the results obtained by the proposed method and the known spectrophotometric method confirmed its usefulness for investigation of Fe^2+^-chelating activity of different compounds. 
